# Evaluating the roles of microRNAs associated with nonalcoholic fatty liver disease in hepatocellular carcinoma tumorigenesis: a systematic review and network analysis

**DOI:** 10.3389/fmed.2024.1462513

**Published:** 2024-11-15

**Authors:** Qinghua Peng, Xiaoning Zhu, Yuanyuan Jiang, Mengyun Peng, Ding Zheng, Xiaodong Wang, Yoke Kqueen Cheah, Jing Wang

**Affiliations:** ^1^Hepatobiliary Department, The Affiliated Traditional Chinese Medicine Hospital, Southwest Medical University, Luzhou, China; ^2^Sichuan Clinical Research Center for Traditional Chinese Medicine of Liver Diseases, The Affiliated Traditional Chinese Medicine Hospital, Southwest Medical University, Luzhou, China; ^3^Department of Biomedical Science, Faculty of Medicine and Health Sciences, Universiti Putra Malaysia, Serdang, Selangor, Malaysia; ^4^UPM-MAKNA Cancer Research Laboratory, Institute of Bioscience, Universiti Putra Malaysia, Serdang, Selangor, Malaysia

**Keywords:** nonalcoholic fatty liver disease, hepatocellular carcinoma, microRNAs, biological functions, signaling pathway, systematic review

## Abstract

**Introduction:**

Non-alcoholic fatty liver disease (NAFLD) has become the leading cause of chronic liver disease worldwide. Hepatocellular carcinoma (HCC) is the fifth most common malignancy worldwide, with high morbidity and mortality. The rapidly increasing incidence of NAFLD is becoming an essential precursor of HCC globally. MicroRNAs (miRNAs) are involved in the progression of NAFLD and HCC.

**Method:**

Potential miRNAs associated with NAFLD in HCC tumorigenesis were identified through a systematic review, and their roles were evaluated by data mining analysis. The biological function of the potential miRNA and its target genes in NAFLD and HCC were evaluated by bioinformatic analysis.

**Result:**

MIR122 was identified as the potential miRNA associated with NAFLD and HCC. Then, MIR122 expression was significantly lower in HCC patients, and higher MIR122 levels were associated with significantly better overall survival. Next, the biological functions of MIR122 and target genes were predicted to be involved in inflammation, fibrosis, cell proliferation, invasion, metastasis, and apoptosis. In particular, the FOXO signaling pathway may regulate the above biological functions.

**Conclusion:**

MIR122 was suggested to be involved in progressing from NAFLD to HCC through the PI3K/AKT/FOXO pathway.

**Systematic review registration:**

PROSPERO, identifier: CRD 42024517940.

## 1 Introduction

Nonalcoholic fatty liver disease (NAFLD) is characterized by the histological presence of steatosis in more than 5% of hepatocytes without alcohol abuse (1). Metabolic syndrome is the primary risk factor for NAFLD, which is characterized by obesity, hyperglycemia, dyslipidemia, and systemic hypertension (HTN) (2). The spectrum of NAFLD encompasses nonalcoholic fatty liver (NAFL), nonalcoholic steatohepatitis (NASH), and their related fibrosis, cirrhosis, and hepatocellular carcinoma (HCC) (3). The global prevalence of NAFLD is estimated to be around 25.2%, comprising 27.4% in Asia, with the highest being in China at over 33%, and its incidence and prevalence are escalating worldwide (4, 5).

More than 80% of cases of Primary Liver cancer (PLC) are HCC, which stands as the fifth most common cancer globally and is the second most mortality malignancy (6). Risk factors for HCC include hepatitis B and C, exposure to dietary toxins, alcohol abuse, and metabolic liver diseases (7). The annual incidence of HCC in NASH patients with cirrhosis is estimated to range from 0.5 to 2.6%, and the rate in non-cirrhotic patients is approximately 0.01% to 0.13% (5). During the obesity epidemic, the incidence of NAFLD-related HCC is experiencing a pronounced and exponential increase (8). NAFLD is becoming an essential precursor of HCC worldwide (9). However, the molecular mechanism between NAFLD and HCC has not been thoroughly studied until now.

MicroRNAs (miRNAs) are a subtype of small, endogenous, noncoding RNAs with lengths typically ranging from 19 to 25 nucleotides. MiRNAs exert their regulatory influence by inversely modulating gene expression through direct induction of messenger RNA (mRNA) degradation or suppressing translation via base pairing with complementary sites in target mRNAs' 3′-untranslated regions (3′-UTRs) (10, 11). Due to this feature, miRNAs have been identified to play critical post-transcription roles in many diseases, including NAFLD and HCC (12). As reported, miRNAs regulate tumor genesis and progression in HCC, such as proliferation, invasion, recurrence, and metastasis (13). Meanwhile, miRNAs also have crucial roles in regulating NAFLD, including the pathological process of steatosis and inflammation in liver cells (13). However, the co-regulated roles of miRNAs between NAFLD and HCC remain unclear.

In this study, we aimed to evaluate the roles of miRNAs associated with NAFLD in HCC tumorigenesis through systematic review and data mining analysis, and the mechanism was evaluated by bioinformatic analysis.

## 2 Materials and methods

### 2.1 System review

A systematic review proceeded for the research objective by following the Preferred Reporting Items for Systematic Reviews and Meta-Analysis (PRISMA) statement. This system review has been officially registered in the Prospective Registry of International Systematic Reviews (PROSPERO) database (CRD 42024517940).

### 2.2 Search strategy

The researchers searched four public databases (PubMed, EMBASE, Cochrane, and Web of Science) from their creation to January 2024. The search strategy was constructed around the PICOS tool: (P) Population: Patients with NAFLD, (I) Intervention: miRNA, (C) Comparison: Significant high or low expression of miRNAs, (O) Outcomes: Progression of steatosis or fibrosis in population with NAFLD, (S) Study type: Comparative research. The complete detailed search strategy is shown in Table 1.

**Table 1:** Search strategy in PubMed, Embase, Cochrane, and WOS.

**S. No**.	**Search terms**	**Search results**
1.Pubmed	((((((((((((((Non-alcoholic Fatty Liver Disease[MeSH Terms]) OR (Nonalcoholic Fatty Liver Disease[MeSH Terms])) OR (NAFLD)) OR (Nonalcoholic Fatty Liver Disease)) OR (Fatty Liver, Nonalcoholic)) OR (Fatty Livers, Nonalcoholic)) OR (Liver, Nonalcoholic Fatty)) OR (Livers, Nonalcoholic Fatty)) OR (Nonalcoholic Fatty Liver)) OR (Nonalcoholic Fatty Livers)) OR (Nonalcoholic Steatohepatitis)) OR (Nonalcoholic Steatohepatitides)) OR (Steatohepatitides, Nonalcoholic)) OR (Steatohepatitis, Nonalcoholic)) AND ((((((((((((((((((MicroRNAs[MeSH Terms]) OR (MicroRNA[MeSH Terms])) OR (miRNAs)) OR (Micro RNA)) OR (RNA, Micro)) OR (miRNA)) OR (Primary MicroRNA)) OR (MicroRNA, Primary)) OR (Primary miRNA)) OR (miRNA, Primary)) OR (pri-miRNA)) OR (pri miRNA)) OR (RNA, Small Temporal)) OR (Temporal RNA, Small)) OR (stRNA)) OR (Small Temporal RNA)) OR (pre-miRNA)) OR (pre miRNA))	880
2.Embase	#1“Non-alcoholic Fatty Liver Disease”:ab,kw OR “non alcoholic fatty liver disease”:ab,kw OR “NAFLD”:ab,kw OR “Nonalcoholic Fatty Liver Disease”:ab,kw OR “Fatty Liver, Nonalcoholic”:ab,kw OR “Fatty Livers, Nonalcoholic”:ab,kw OR “Liver, Nonalcoholic Fatty”:ab,kw OR “Livers, Nonalcoholic Fatty”:ab,kw OR “Nonalcoholic Fatty Liver”:ab,kw OR “Nonalcoholic Fatty Livers”:ab,kw OR “Nonalcoholic Steatohepatitis”:ab,kw OR “Nonalcoholic Steatohepatitides”:ab,kw OR “Steatohepatitides, Nonalcoholic”:ab,kw OR “Steatohepatitis, Nonalcoholic”:ab,kw #2“MicroRNAs”:ab,kw OR “MicroRNA”:ab,kw OR “miRNAs”:ab,kw OR “Micro RNA”:ab,kw OR “RNA, Micro”:ab,kw OR “miRNA”:ab,kw OR “Primary MicroRNA”:ab,kw OR “MicroRNA, Primary”:ab,kw OR “Primary miRNA”:ab,kw OR “miRNA, Primary”:ab,kw OR “pri-miRNA”:ab,kw OR “pri miRNA”:ab,kw OR “RNA, Small Temporal”:ab,kw OR “MicroRNAs”:ab,kw OR “Temporal RNA, Small”:ab,kw OR “stRNA”:ab,kw OR “Small Temporal RNA”:ab,kw OR “pre-miRNA”:ab,kw OR “pre miRNA”:ab,kw #1 and #2	1,037
3.Cochrane	#1 MeSH descriptor: [Non-alcoholic Fatty Liver Disease] explode all trees #2 (Non-alcoholic Fatty Liver Disease or non alcoholic fatty liver disease or NAFLD or Nonalcoholic Fatty Liver Disease or Fatty Liver, Nonalcoholic or Fatty Livers, Nonalcoholic or Liver, Nonalcoholic Fatty or Livers, Nonalcoholic Fatty or Nonalcoholic Fatty Liver or Nonalcoholic Fatty Livers or Nonalcoholic Steatohepatitis or Nonalcoholic Steatohepatitides or Steatohepatitides, Nonalcoholic or Steatohepatitis, Nonalcoholic):ti,ab,kw (Word variations have been searched) #3 #1 or #2 #4 MeSH descriptor: [MicroRNAs] explode all trees #5 (MicroRNA or miRNAs or Micro RNA or RNA, Micro or miRNA or Primary MicroRNA or MicroRNA, Primary or Primary miRNA or miRNA, Primary or pri-miRNA or pri miRNA or RNA, Small Temporal or MicroRNAs or Temporal RNA, Small or stRNA or Small Temporal RNA or pre-miRNA or pre miRNA):ti,ab,kw (Word variations have been searched) 1,819 #6 #4 or #5 #7 #3 and #6	26
4.WOS	TS = (MicroRNAs OR MicroRNA OR miRNAs OR MicroRNA OR RNA, Micro OR miRNA OR Primary MicroRNA OR MicroRNA, Primary OR Primary miRNA OR miRNA, Primary OR pri-miRNA OR pri miRNA OR RNA, Small Temporal OR Temporal RNA, Small OR stRNA OR Small Temporal RNA OR pre-miRNA OR pre miRNA) AND TS = (Non-alcoholic Fatty Liver Disease OR Non alcoholic Fatty Liver Disease OR NAFLD OR Nonalcoholic Fatty Liver Disease OR Fatty Liver, Nonalcoholic OR Fatty Livers, Nonalcoholic OR Liver, Nonalcoholic Fatty OR Livers, Nonalcoholic Fatty OR Nonalcoholic Fatty Liver OR Nonalcoholic Fatty Livers OR Nonalcoholic Steatohepatitis OR Nonalcoholic Steatohepatitides OR Steatohepatitides, Nonalcoholic OR Steatohepatitis, Nonalcoholic)	1,415

### 2.3 Eligibility criteria

The inclusion criteria were as follows: (1) Experimental group with altered miRNA as an intervention for people with NAFLD, (2) Control group with significantly high or low expression of miRNAs, (3) Cohort studies, (4) Outcome included the progression of steatosis, or fibrosis in population with NAFLD.

The exclusion criteria were as follows: (1) non-human studies, (2) non-comparative studies, (3) reviews and systematic evaluations meta-analyses, and (4) case reports, reviews, letters, and editorials.

### 2.4 Study selection

The articles underwent screening and exclusion using the Rayyan website. Firstly, two researchers (Y. Jiang and H Peng) screened the literature titles to identify duplicates, non-randomized controlled trial studies, reviews, systematic evaluations, meta-analyses, case reports, letters, and editorials. Then, the same two researchers (Y. Jiang and H Peng) reviewed the literature's abstracts to determine inclusion and exclusion criteria. Finally, both researchers (Y. Jiang and H Peng) independently read the full articles of the selected ones and further refined the inclusion criteria. Throughout this process, the researchers worked independently, and disputes were resolved by the third author (X Zhu).

### 2.5 Data mining and bioinformatic analysis

To identify the potential miRNAs associated with HCC tumorigenesis, we employed the COREMINE database (https://www.coremine.com/), which utilizes a text-mining algorithm. Searching keywords, including “Liver neoplasms” and “Cell Transformation, Neoplastic,” were selected in COREMINE to compile a list of microRNAs linked to HCC tumorigenesis.

The difference in expression and prognosis efficacy of miRNA in HCC patients was investigated in the ENCORI database (https://rnasysu.com/encori/) and Kaplan-Meier plotter (https://kmplot.com/analysis/). The miRNA targets and their biological roles were identified using the ONCO.IO database (https://onco.io/). Next, a regulatory network model was constructed to understand miRNA-target interactions better. The Enrichr database (https://amp.pharm.mssm.edu/Enrichr/) enriched the significant pathways related to the miRNA target genes.

### 2.6 Statistical analysis

Statistical analysis followed the specific rules of the COREMINE database, Onco.io database, Kaplan–Meier plotter, and Enrichr database online tools. In the Onco.io and Enrichr databases, *P* < 0.05 was considered statistically significant. In the COREMINE database, Extracted Associations were set as co-mentioned with all selected nodes, and *P* < 0.05.

## 3 Results

### 3.1 Study characteristics

**Figure 1:**
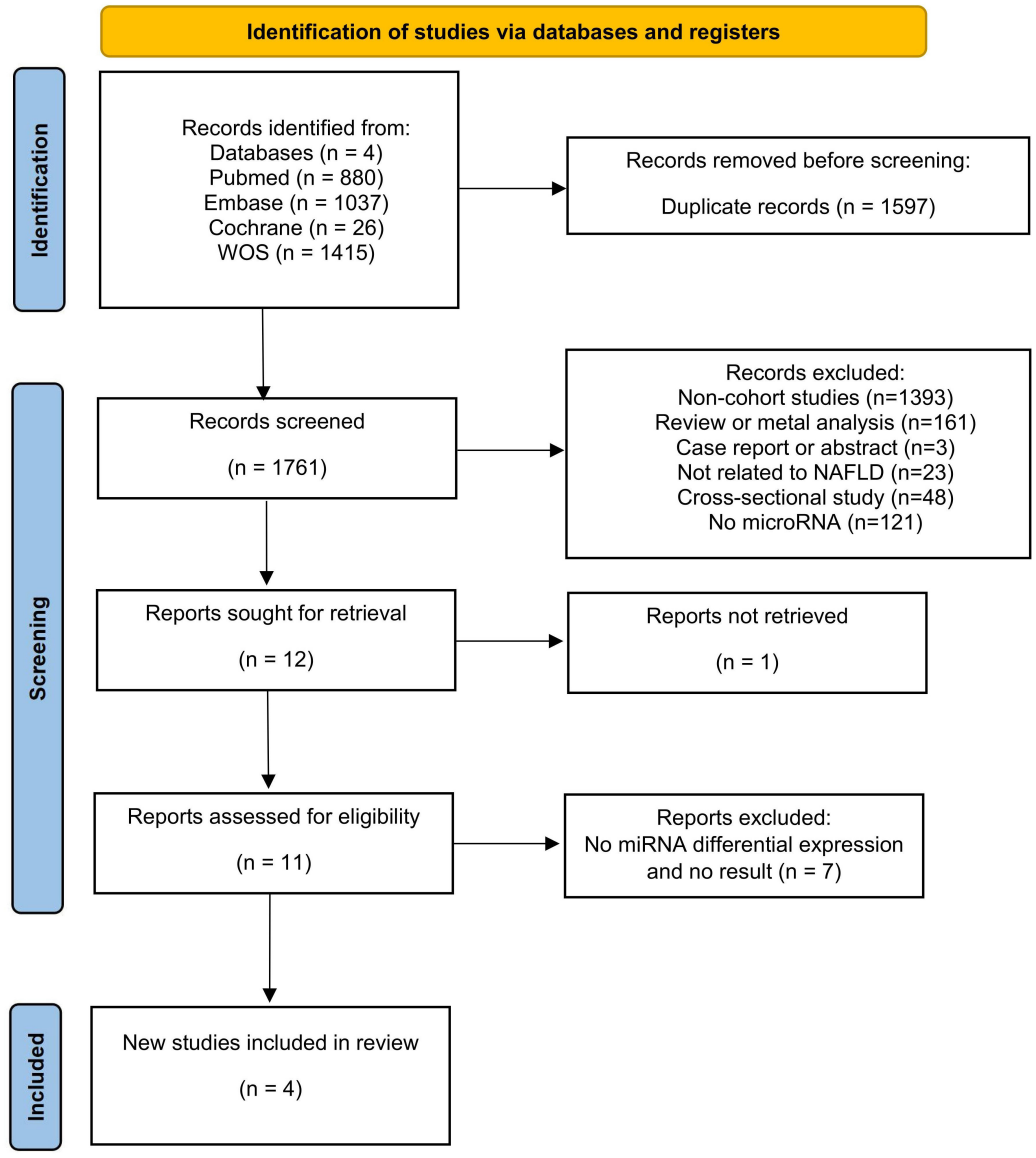
Flow diagram for the selection process of the systematic review.

The literature search yielded 3,358 articles (PubMed = 880, Web of Science = 1,415, Embase = 1,037, Cochrane = 26), with 1,597 identified as duplicates and subsequently removed. Among the remaining 1,761 articles, 1,749 abstracts were reviewed and removed based on the inclusion and exclusion criteria. Then, one of the remaining 12 articles was excluded because the study was not retrieved. After that, 11 articles underwent thorough full-text reading, excluding eight due to a lack of differentially expressed miRNAs and non-compliance with outcome metrics. Four articles met the criteria and were included in the current review (Figure 1).

The four studies included 863 patients who were diagnosed with NAFLD by liver biopsy (14–17). Among them, 21 patients died during the follow-up period, and 13 developed HCC. Serum microRNA 122 (MIR122) levels tend to decrease before progression to the fibrotic stage, and reduced levels may reflect an increased risk of hepatocellular carcinoma. Serum MIR122 and a history of hepatocellular carcinoma are independent poor prognostic factors. Detailed characteristics of the included studies are shown (Table 2).

**Table 2:** Detailed characteristics of the included studies.

**References**	**Country**	**Number of patients (male, female)**	**Follow up**	**Type of evidence**	**miRNA**	**Expression**
Akuta et al. (14)	Japan	305 (178, 127)	35.3 years	Lower MIR122 levels of serum is link to the HCC and progression of fibrosis in NASH patients	MIR122	Down (Serum)
Akuta et al. (15)	Japan	36 (20, 16)	19.0 years	Lower MIR122 levels of serum is related to the histopathological progression of NAFLD patient	MIR122	Down (Serum)
Akuta et al. (16)	Japan	441 (266, 175)	42.9 years	Serum MIR122miR122 and FIB-4 are the risk factors for mortality in NAFLD patients	MIR122	Down (Serum)
Akuta et al. (17)	Japan	81 (47, 34)	23.5 years	Serum MIR122 is a prediction factor of the prognosis for NAFLD patients with severe fibrosis stage and no improvement of the stage scores	MIR122	Down (Serum)

### 3.2 MIR122 is the potential miRNA associated with NAFLD and tumor genesis of HCC

**Figure 2:**
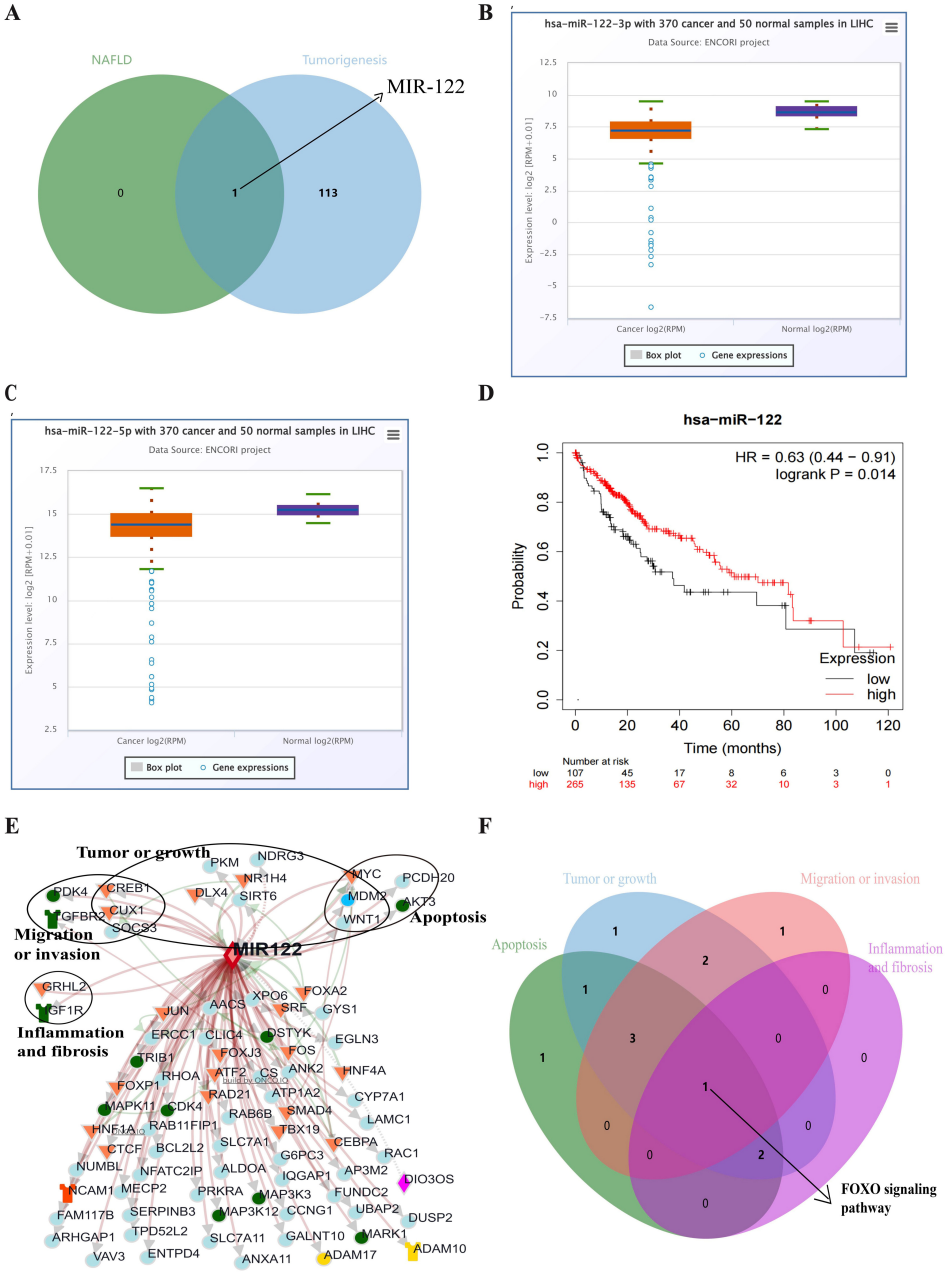
**(A)** Venn diagram showing all common microRNAs in both pathways. **(B)** The Starbase online website analyzes MIR122-3p's relative expression in HCC. **(C)** The Starbase online website analyzes MIR122-5p's relative expression in HCC. **(D)** The 10-year survival rate of the MIR122 low-expression group decreased dramatically. **(E)** A network of MIR122 and its target genes. **(F)** Target signaling pathways associated with biological processes.

One hundred fourteen miRNAs related to liver tumors and tumor cell transformation were identified from the COREMINE database (*P* < 0.05). Subsequently, MIR122 was detected as involved in the NAFLD progression and HCC tumorigenesis (Figure 2A). The expression of MIR122 decreased in HCC patients, and the 5-year overall survival rate in the group with high MIR122 expression was significantly higher than in the group with lower expression (*P* = 0.014; Figures 2B–D).

### 3.3 The potential biological roles of the target genes of MIR122 in cancer

The ONCO.IO database was used to obtain the regulatory network of MIR122 and its target genes in cancer. There are 80 target genes, including 13 upregulated and 67 downregulated genes (Table 3). The results showed that MIR122 interacted significantly and formed a target gene network (Figure 2E). These target genes significantly regulate essential biological processes such as inflammation, fibrosis, tumor growth, migration, and apoptosis. Some targets, such as DLX4, NDRG3, WNT1, MYC, CREB1, and SIRT6, are involved in tumor or cell proliferation. Some other targets are involved in inflammation and fibrosis, such as TGFBR2 and GRHL2. In addition, some targets co-regulate the same biological processes. For example, IGF1R, PDK4, and SOCS3 are involved in tumor proliferation, migration, and invasion. MYC and MDM2 are involved in inflammation and fibrosis and are associated with tumor proliferation. The roles of MIR122 target genes in regulating these biological processes are listed in Table 4.

**Table 3:** A list of microRNAs' targets based on relative position.

**Regulations**	**Genes**
Upregulated	HNF4A, HNF1A, FOXA2, CEBPA, JUN, ATF2, FOS, MYC, SIRT6, NR1H4, GRHL2, SMAD4, DIO3OS
Downregulated	ALDOA, RHOA, ADAM17, RAC1, CCNG1, CUX1, MAP3K12, LAMC1, MAP3K3, CLIC4, BCL2L2, VAV3, CTCF, MARK1, RAD21, ANK2, NFATC2IP, ENTPD4, ANXA11, RAB6B, RAB11FIP1, FOXP1, MECP2, NCAM1, UBAP2, TBX19, AACS, DUSP2, ATP1A2, MAPK11, FUNDC2, AKT3, TPD52L2, GALNT10, G6PC3, AP3M2, XPO6, FOXJ3, SLC7A11, TRIB1, EGLN3, NUMBL, DSTYK, FAM117B, IGF1R, SRF, ADAM10, WNT1, SLC7A1, CYP7A1, GYS1, PKM, ARHGAP1, IQGAP1, CDK4, NDRG3, PCDH20, MDM2, CS, PDK4, CREB1, DLX4, SOCS3, TGFBR2, SERPINB3, ERCC1, PRKRA

**Table 4:** Overview of biological processes in Mir-122 and its target genes.

**Process**	**Number**	**Gene**
Tumor or cell growth	13	DLX4, NDRG3, WNT1, MYC, CREB1, SIRT6, PKM, MDM2, NR1H4, IGF1R, PDK4, SOCS3, TGFBR2
Migration or invasion	5	IGF1R, PDK4, SOCS3, CUX1, CREB1
Apoptosis	5	MDM2, PCDH20, AKT3, WNT1, MYC
Inflammation and fibrosis	2	TGFBR2, GRHL2
TGF-beta signaling	1	TGFBR2
JAK/STAT signaling	1	SOCS3

The potential pathways of target genes were further investigated using the Enrichr database. These biological processes involve common signaling pathways, such as the PI3K-Akt signaling pathway, FoxO signaling pathway, TGF-beta signaling pathway, MAPK signaling pathway, and JAK-STAT signaling pathway. The FoxO signaling pathway involves all biological processes (Figure 2F).

## 4 Discussion

MiRNAs are post-transcriptional regulators that play essential roles in various processes, and their dysregulation has been associated with NAFLD and HCC (18, 19). For example, miRNAs can target multiple genes involved in developing NAFLD, including cholesterol biosynthesis, *de novo* lipogenesis, hepatic energy metabolism, inflammation, cell regeneration, and fibrotic signaling (13, 20). Adipokines secreted by adipose tissue tend to induce systemic chronic inflammation, which increases the risk of various types of carcinogenesis (21). MiRNAs play an essential role in the progression from NAFLD to HCC by regulating the expression of key signaling pathways and targets (21). Thus, dysregulated miRNAs serve as a bridge linking NAFLD and HCC, such as MIR122. Meanwhile, chronic liver inflammation of different etiologies, including NAFLD, is associated with reduced MIR122 expression in hepatocytes, and MIR122 expression levels are relatively low in NAFLD-related HCC compared to healthy tissues (22–24).

The involvement of MIR122 has been reported to be a tumor suppressor in inflammation, fibrosis, cancer cell proliferation, invasion, metastasis, and apoptosis (25). We detected that the FOXO signaling pathway collectively participates in these biological processes, and MIR122 target genes are mainly concentrated in the FOXO signaling pathway (26, 27). FOXO proteins are growth and stress-regulated transcription factors, which play essential roles in glucose and lipid metabolism and are involved in various cellular functions (28–32). FOXO family members, FOXO1, FOXO3a, and FOXO4, can be phosphorylated by AKT, leading to loss of FOXO DNA-binding activity. Inactivation of FOXO1 and FOXO3 also results in hypertriglyceridemia and hypercholesterolemia due to increased hepatic lipid secretion and mild steatosis, indicating that FOXO1 and FOXO3 work together to inhibit critical pathways in adipogenesis as well as enhance gluconeogenic genes (33). In addition, it has been shown that FOXO1 increases MIR122 promoter activity by binding to the MIR122 promoter, which can be inhibited by leptin-induced phosphorylation of FOXO1 by the PI3K/Akt signaling pathway, and it was demonstrated that FOXO1 attenuates leptin-associated HSC activation and hepatic fibrosis in the ob/ob mouse model (34). MIR122 also directly inhibits FOXO3 to promote the development of NAFLD (35).

FOXO1 is the primary member of the FOXO family that can promote HCC tumorigenesis and progression (36). FOXO family members can be regulated by the PI3K/Akt pathway and act as a vital target of the insulin/insulin-like growth factor (IGF)-1 signaling pathway to regulate cellular functions (37–39). Studies have shown that FOXO isoforms exhibit differential expression in HCC's primary tumor tissues and cells (36, 40, 41).

In HCC patients, increased FOXO1 expression was found to predict a favorable prognosis for HCC patients and was negatively correlated with vascular infiltration (41). FOXO1 is essential in multiple signaling pathways, especially the insulin/PI3K/AKT signaling pathway, which negatively regulates FOXO1 (42, 43). Activation of this pathway increases cell survival, promotes cell proliferation, and induces cancer development (44, 45). In HCC, overactive Akt signaling inhibits the transcriptional activity of FOXO1, weakening the defense against oxidative stress. FOXO1 typically inhibits the expression of epithelial-mesenchymal transition (EMT)-inducing transcription factors and transforming growth factor-beta (TGF-β), leading to a subsequent increase in EMT and HCC cell migration and invasion (46, 47).

This study has some limitations. First, considering the long period of progression from NAFLD to HCC and acknowledging that steatosis and fibrosis are crucial factors for NAFLD progression to HCC, we set the search strategy of the potential miRNAs as one that can significantly change during the progression of steatosis or fibrosis in a population with NAFLD. Except for MIR122, other relevant miRNAs with significant expression differences were not identified in this systematic review after searching multiple databases due to a lack of cohort data in NAFLD studies. The second limitation is that the population of the included studies was limited to Japanese patients. On the one hand, this is one of the few groups that have conducted long-term follow-up cohort studies on NAFLD patients. On the other hand, other studies found that MIR122 levels are significantly lower in NASH patients in Western populations and that MIR122 expression decreases significantly with the progression of hepatic steatosis (23, 48). Additionally, MIR122 levels were found to be downregulated in the serum of HCC patients from Egypt (49). These results supported evidence that MIR122 may be the potential bridging factor between NAFLD and HCC. However, the value of the MIR122's biomarker and treatment target must still be determined through the long-term cohort study and experiment. Nevertheless, low expression of MIR122 is associated with a poor prognosis in patients with NAFLD-associated HCC, providing a new view for discovering new biomarkers and their associated targets and pathways through systematic review and data mining approaches.

## 5 Conclusions

In summary, MIR122 may be a potential regulator correlated to NAFLD and HCC and involved in the process of NAFLD to HCC through the PI3K/AKT/FOXO pathway. However, the specific mechanism needs further experimental verification.

## Data Availability

Publicly available datasets were analyzed in this study. This data can be found at: COREMINE database (https://www.coremine.com/) ENCORI database (https://rnasysu.com/encori/) Kaplan–Meier plotter (https://kmplot.com/analysis/) ONCO.IO database (https://onco.io/) Enricher database (https://amp.pharm.mssm.edu/Enrichr/).
