# Supplementary material for: Evaluating the roles of microRNAs associated with nonalcoholic fatty liver disease in hepatocellular carcinoma tumorigenesis: a systematic review and network analysis
Source: Front Med (Lausanne). 2024 Nov 15;11:1462513. doi: 10.3389/fmed.2024.1462513 (PMC11604432; doi:10.3389/fmed.2024.1462513)
Supplement: Supplementary file 1 [file Table_1.DOCX]

**Supplementary Material(S-1)**

**Table S-1: List of the gene abbreviations that appear in the manuscript.**

| **LIST OF THE GENE ABBREVIATIONS** | |
| --- | --- |
| HNF4A | Hepatocyte Nuclear Factor 4 Alpha |
| HNF1A | HNF1 Homeobox A |
| FOXA2 | Forkhead Box A2 |
| CEBPA | CCAAT Enhancer Binding Protein Alpha |
| JUN | Jun Proto-oncogene, AP-1 Transcription Factor Subunit |
| ATF2 | Activating Transcription Factor 2 |
| FOS | Fos Proto-oncogene, AP-1 Transcription Factor Subunit |
| MYC | MYC Proto-oncogene, bHLH Transcription Factor |
| SIRT6 | Sirtuin 6 |
| NR1H4 | Nuclear Receptor Subfamily 1 Group H Member 4 |
| GRHL2 | Grainyhead Like Transcription Factor 2 |
| SMAD4 | SMAD Family Member 4 |
| DIO3OS | DIO3 Opposite Strand Upstream RNA |
| ALDOA | Aldolase, Fructose-bisphosphate A |
| RHOA | Ras Homolog Family Member A |
| ADAM17 | ADAM Metallopeptidase Domain 17 |
| RAC1 | Rac Family Small GTPase 1 |
| CCNG1 | Cyclin G1 |
| CUX1 | Cut Like Homeobox 1 |
| MAP3K12 | Mitogen-activated Protein Kinase Kinase Kinase 12 |
| LAMC1 | Laminin Subunit Gamma 1 |
| MAP3K3 | Mitogen-activated Protein Kinase Kinase Kinase 3 |
| CLIC4 | Chloride Intracellular Channel 4 |
| BCL2L2 | BCL2 Like 2 |
| VAV3 | Vav Guanine Nucleotide Exchange Factor 3 |
| CTCF | CCCTC-binding Factor |
| MARK1 | Microtubule Affinity Regulating Kinase 1 |
| RAD21 | RAD21 Cohesin Complex Component |
| ANK2 | Ankyrin 2 |
| NFATC2IP | Nuclear Factor Of Activated T Cells 2 Interacting Protein |
| ENTPD4 | Ectonucleoside Triphosphate Diphosphohydrolase 4 |
| ANXA11 | Annexin A11 |
| RAB6B | RAB6B, Member RAS Oncogene Family |
| RAB11FIP1 | RAB11 Family Interacting Protein 1 |
| FOXP1 | Forkhead Box P1 |
| MECP2 | Methyl-CpG Binding Protein 2 |
| NCAM1 | Neural Cell Adhesion Molecule 1 |
| UBAP2 | Ubiquitin Associated Protein 2 |
| TBX19 | T-box Transcription Factor 19 |
| AACS | Acetoacetyl-CoA Synthetase |
| DUSP2 | Dual Specificity Phosphatase 2 |
| ATP1A2 | ATPase Na+/K+ Transporting Subunit Alpha 2 |
| MAPK11 | Mitogen-activated Protein Kinase 11 |
| FUNDC2 | FUN14 Domain Containing 2 |
| AKT3 | AKT Serine/Threonine Kinase 3 |
| TPD52L2 | TPD52 like 2 |
| GALNT10 | Polypeptide N-acetylgalactosaminyltransferase 10 |
| G6PC3 | Glucose-6-phosphatase Catalytic Subunit 3 |
| AP3M2 | Adaptor Related Protein Complex 3 Subunit Mu 2 |
| XPO6 | Exportin 6 |
| FOXJ3 | Forkhead Box J3 |
| SLC7A11 | Solute Carrier Family 7 Member 11 |
| TRIB1 | Tribbles Pseudokinase 1 |
| EGLN3 | Egl-9 Family Hypoxia Inducible Factor 3 |
| NUMBL | NUMB Like Endocytic Adaptor Protein |
| DSTYK | Dual Serine/Threonine And Tyrosine Protein Kinase |
| FAM117B | Family With Sequence Similarity 117 Member B |
| IGF1R | Insulin Like Growth Factor 1 Receptor |
| SRF | serum response factor |
| ADAM10 | ADAM Metallopeptidase Domain 10 |
| WNT1 | Wnt Family Member 1 |
| SLC7A1 | Solute Carrier Family 7 Member 1 |
| CYP7A1 | Cytochrome P450 Family 7 Subfamily A Member 1 |
| GYS1 | Glycogen Synthase 1 |
| PKM | Pyruvate Kinase M1/2 |
| ARHGAP1 | Rho GTPase Activating Protein 1 |
| IQGAP1 | IQ motif containing GTPase activating protein 1 |
| CDK4 | Cyclin Dependent Kinase 4 |
| NDRG3 | NDRG family member 3 |
| PCDH20 | Protocadherin 20 |
| MDM2 | MDM2 Proto-oncogene |
| CS | Citrate Synthase |
| PDK4 | Pyruvate Dehydrogenase Kinase 4 |
| CREB1 | cAMP Responsive Element Binding Protein 1 |
| DLX4 | Distal-less Homeobox 4 |
| SOCS3 | Suppressor Of Cytokine Signaling 3 |
| TGFBR2 | Transforming Growth Factor Beta Receptor 2 |
| SERPINB3 | Serpin Family B Member 3 |
| ERCC1 | ERCC Excision Repair 1, Endonuclease Non-catalytic Subunit |
| PRKRA | Protein Activator Of Interferon Induced Protein Kinase EIF2AK2 |
